# A Prospective Study of Liver Regeneration After Radiotherapy Based on a New (Su’S) Target Area Delineation

**DOI:** 10.3389/fonc.2021.680303

**Published:** 2021-08-26

**Authors:** Ting-Shi Su, Li-Qing Li, Shi-Xiong Liang, Bang-De Xiang, Jian-Xu Li, Jia-Zhou Ye, Le-Qun Li

**Affiliations:** ^1^Department of Radiation Oncology, Guangxi Medical University Cancer Hospital, Nanning, China; ^2^Department of Hepatobiliary Surgery, Guangxi Medical University Cancer Hospital, Nanning, China

**Keywords:** radiotherapy, liver regeneration, model, nomogram, hepatocellular carcinoma

## Abstract

**Background:**

In this study, we designed a new (Su’S) target area delineation to protect the normal liver during liver regeneration and prospectively evaluate liver regeneration after radiotherapy, as well as to explore the clinical factors of liver regeneration and established a model and nomogram.

**Methods:**

Thirty patients treated with preoperative downstaging radiotherapy were prospectively included in the training cohort, and 21 patients treated with postoperative adjuvant radiotherapy were included in the validation cohort. The cut-off points of each optimal predictor were obtained using receiver-operating characteristic analysis. A model and nomogram for liver regeneration after radiotherapy were developed and validated.

**Results:**

After radiotherapy, 12 (40%) and 13 (61.9%) patients in the training and validation cohorts experienced liver regeneration, respectively. The risk stratification model based on the cutoffs of standard residual liver volume spared from at least 20 Gy (SVs20 = 303.4 mL/m^2^) and alanine aminotransferase (ALT=43 u/L) was able to effectively discriminate the probability of liver regeneration. The model and nomogram of liver regeneration based on SVs20 and ALT showed good prediction performance (AUC=0.759) in the training cohort and performed well (AUC=0.808) in the validation cohort.

**Conclusions:**

SVs20 and ALT were optimal predictors of liver regeneration. This model may be beneficial to the constraints of the normal liver outside the radiotherapy-targeted areas.

## Introduction

Hepatocellular carcinoma (HCC) is the fourth most common cause of cancer-related deaths worldwide (1). Advances in technology enable more accurate and effective radiotherapy (RT), while clinical exploration continues to expand the indications for radiotherapy beyond the formal paradigm of HCC. External beam radiotherapy has been used as a palliative or radical treatment depending on the stage of HCC (2–6). In the latest EASL-EORTC Clinical Practice Guidelines and National Comprehensive Cancer Network Guidelines, radiotherapy is recommended as an alternative locoregional therapy for potentially resectable and unresectable HCC (7, 8). In particular, a multidisciplinary team approach involving radiotherapy is more frequently adopted for selected patients in China and Southeast Asia (9–12).

In the past, regarding the clinical practice of radiotherapy for liver cancer, more attention has been paid to the prevention and treatment of radiation-induced liver injury (13–15), but no in-depth study on liver regeneration has been conducted. Liver regeneration after hepatectomy (16), associated with liver partition and portal vein ligation for staged hepatectomy (ALPPS) (17) and portal vein embolization (PVE) (18) were beneficial to the recovery of treatment-induced liver injury. With the gradual application of preoperative and postoperative radiotherapy for HCC (19–23), liver regeneration after radiotherapy will become a new focus of clinical attention for the prevention or recovery of radiation-induced liver damage. However, the clinical factors that influence liver regeneration after radiotherapy are poorly understood. Therefore, in this study, we designed a new target area delineation to protect the normal liver of liver regeneration and prospectively evaluate liver regeneration after preoperative and postoperative radiotherapy, and further explored the clinical factors and established a model and nomogram for liver regeneration after radiotherapy for HCC.

## Methods

### Patients

Patients who underwent preoperative downstaging or postoperative radiotherapy for HCC at Guangxi Medical University Cancer Hospital were included in the study. The training cohort included 30 patients treated with radiotherapy for downstaging non-surgical locally HCC before liver resection from 2018-2019 (ChiCTR1800015350). The inclusion criteria for radiotherapy in down-staging HCC were (1): primary local unresectable HCC with macroscopic vascular tumor emboli (2); One to three lesions in a single lobe; surgical resection was expected to be performed if a descending stage of tumor or thrombolysis (3); Child-Pugh-A or B7 class; and (4) Eastern Clinical Oncology Group score 0-1. The exclusion criteria were as follows: (a) prior history of abdominal radiotherapy, (b) intrahepatic cholangiocellular carcinoma, (c) gallbladder metastases, and/or (d) liver metastases.

The validation cohort included 21 patients treated with postoperative adjuvant therapy for HCC with microvascular vascular invasion or narrow margins after hepatectomy from 2017 to 2019 (NCT 02309788). Patients received adjuvant radiotherapy according to the following criteria (1): HCC with no preoperative radiotherapy (2); resectable lesion with narrow margin (less than 1 cm), at the same time retaining a sufficient residual liver tissue to maintain adequate function (3); compensated cirrhosis or no cirrhosis (4); Child-Pugh A class (5); ECOG score 0-1. The exclusion criteria were (1): presence of distant metastasis (2), palliative resection with residual tumor, and (3) non-HCC confirmed by postoperative pathology (4). Liver failure or decompensation occurs after the surgery.

### Su’S Radiotherapy Target Area Delineation Promotes Liver Regeneration

#### Downstaging Radiotherapy Group

**Figure 1:**
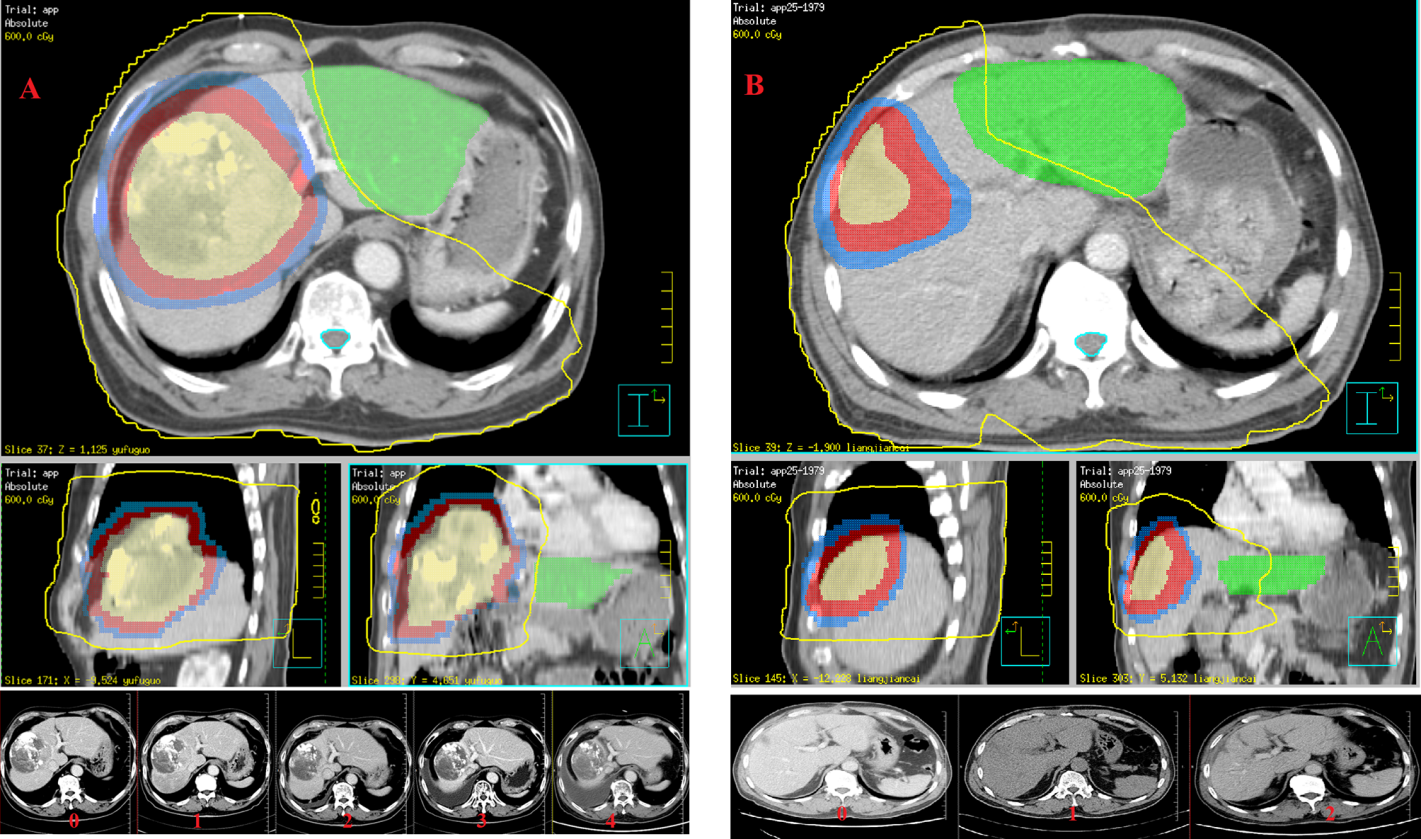
Su’S radiotherapy target area delineation promotes liver regeneration and evaluation of liver regeneration after radiotherapy: **(A)** the training cohort (GTVi, PGTV and PCTV are shown from inside to outside, and the green part represents liver protected volume in preoperative downstaging radiotherapy plan); and **(B)** the testing cohort (GTVtb, PCTV1 and PCTV2 are shown from inside to outside, and the green part represents liver protected volume in postoperative adjuvant radiotherapy plan).

Gross tumor volume (GTV) was defined as intrahepatic tumors and venous tumor thrombus. InterGTV (GTVi) was defined as a 1cm GTV retraction, with the aim of receiving a higher radiation dose to overcome the radiation tolerance caused by central tumor ischemia or hypoxia. The clinical target volume (CTV) was obtained by adding a 0.5 cm margin to the GTV. The planning target volume (PTV) was defined as the CTV and GTV expansion 0.5 cm in horizontal direction and 0.5-0.8 cm head and foot direction for setup uncertainty and respiratory motion. GTV/GTVi areas should avoid more than 1 cm when encountering gastrointestinal organs. The absolute normal liver volume was calculated as the total liver minus the GTV. Liver protected volume was defined as a normal liver segment 2.0 cm away from the CTV, and the purpose of liver protection was to promote liver regeneration (**Figure 1A**). The final radiation dose delivered to the isocentric was 66 Gy for GTVi (4.4 or 3.3 Gy/fx), 60 Gy for PGTV (4.0 or 3.0 Gy/fx), and 45–50 Gy for PCTV (3.0-2.5 Gy/fx) with 15 or 20 fractions (5 fractions per week).

#### Postoperative Adjuvant Radiotherapy Group

Adjuvant radiotherapy was started 4-6 weeks after surgery. The postoperative tumor bed area was designated as GTVtb. The CTV1 extends 0.5 cm on the basis of GTVtb. CTV2 extends 0.5 cm on the basis of CTV1. PTV was defined as the CTV1 and CTV2 expansion 0.5 cm in horizontal direction and 0.5-0.8 cm head and foot direction for setup uncertainty and respiratory motion. GTVtb/CTV1 areas should avoid more than 0.5-1.0 cm when encountering gastrointestinal organs. The absolute normal liver volume was calculated as the total liver minus CTV1. The liver protected volume was defined as a normal liver segment 2.0 cm away from CTV2 (**Figure 1B**), and the purpose of liver protection was to promote liver regeneration. The final radiation dose delivered to the isocentric was 50-60 Gy for PCTV1 (2.0-2.4 Gy/fx), 40-50 Gy for PCTV2 (2.0-2.25 Gy/fx) with 20 or 25 fractions (5 fractions a week).

All target areas were sketched in the MIM 6.8 system (MIM, USA). The Pinnacle 3 system (Philips, Netherlands) was used to accomplish the target area dose planning (YY, USA). All patients were treated with a linear accelerator (ELEKTA Synergy, Sweden and ELEKTA VersaHD, Sweden). There were various dose–volume constraints for the organs at risk. For the liver, the normal liver volume D_mean_< 21 Gy, and normal liver volume spared from at least 10 Gy (V_S_10) was >410 mL and liver protected D_mean_ < 7 Gy. For the stomach, small bowel, and duodenum, D_max_ was < 40–45 Gy each. For the kidneys, V15 was <1/3 V. Similarly, for the spinal cord, D_max_ <40 Gy.

### Liver Regeneration Ratio Assessment

Patients were re-evaluated 1 and 3 months after radiotherapy and every 3-6 months thereafter. Contrast-enhanced CT and/or MRI were performed within 2 weeks before radiotherapy and subsequently at each follow-up visit after radiotherapy. Laboratory examinations assessed the levels of aspartate transaminase (AST), alanine transaminase (ALT), prothrombin time (PT), and levels of albumin, total bilirubin, and alpha-fetoprotein (AFP).

Liver regeneration at each re-evaluation of each patient was performed for comparison. The CT or MRI images before and after radiotherapy were imported into the MIM 6.8, to delineate the hepatic parenchymal volume of the segment and lobe of interest in the same manner. Liver regeneration was defined as an increase of more than 10% of normal liver volume in the areas of the protected hepatic segment or lobe within 1 year after completion of radiotherapy compared to the volume of pre-radiotherapy, and no Child-Pugh class degradation of liver function and tumor progression was observed at the last time. If downstaging surgery or tumor progression occurs in the areas of interest, liver regeneration assessment is discontinued.

### Variable Selection

We screened predictor variables for liver regeneration from the following variables: (a) clinical factors: age, sex, height, weight and hepatitis B virus (HBV) infection; (b) serum biochemical parameters: red blood cells count (RBC), white blood cells count (WBC), hemoglobin (HB), platelets (PLT), total bilirubin (Tbil), albumin, alanine aminotransferase (ALT), aspartate aminotransferase (AST), lactic dehydrogenase (LDH), activated partial thromboplastin time (APTT) and alpha-fetoprotein (AFP); (c) dose-volumetric parameters: functional liver volume, mean dose of the liver (liver-Dmean), GTV volume (the sum of all GTVs), GTV dose and fractions; (d) dosimetric Dataset 1: the percentage of normal protected liver volume (%) spared from at least x Gy (Vx); and (e) dosimetric Dataset 2: the absolute normal liver volume (mL) spared from at least x Gy (Vsx); (f) dosimetric Dataset 3: standard residual normal liver volume (mL/m^2^) spared from at least x Gy (SVsx) (formula: SVsx = Vsx/Body surface area). In the training cohort, the random forest model was applied to rank these factors in descending order of relative importance. In the preliminary screening, factors with an area under a receiver-operating characteristic (ROC) curve (AUC) greater than 0.6, were considered as potential prognostic predictors. Correlation analysis was performed to avoid overfitting. When Spearman’s rho value was greater than 0.65 between the two dosimetric parameters, the one with a lower correlation with liver generation was excluded.

### Calculated Values and Statistical Analysis

Continuous variables were compared using Student’s t-test or the Mann–Whitney U-test, and categorical variables were compared using the chi-square test. The final nomogram was formulated to predict liver regeneration based on prognostic factors extracted from the training cohort. For internal validation, the prediction performance of the nomograms was measured using a calibration curve and concordance index (C-index) to measure internal calibration and discriminative ability. To avoid over-optimism, bootstrap resampling (1000) resamples were applied to correct the C-index for assessment of the nomogram calibration performance. The prognostic model was further validated in a postoperative adjuvant radiotherapy cohort to ascertain its feasibility. The total points of each patient in the testing cohort were calculated according to the established nomogram and used as a predictor of liver regeneration. The AUC of the ROC was used to evaluate the prediction performance of our model using external data.

For additional analyses, the optimal cut-off value for each selected factor of the models was obtained from the Youden’s index in conjunction with the ROC analysis from the testing cohort. Risk stratification was then conducted according to the cut-off point in both the training and validation cohorts, as well as the entire cohort. Fisher’s exact test was used to compare the proportion of patients with liver regeneration between the subgroups. All statistical analyses were completed using R version 4.0.2 (2020–06–22), and a P value less than 0.05, was considered statistically significant.

## Results

### Baseline Characteristics

Fifty-one patients with HCC were included. The study population was predominantly male (n= 46, 90.1%), with a median age of 48 years (range, 21-70 years). Most of the patients (n=37, 72.5%) were infected with CHB.

The baseline characteristics and dosimetric data of the training (n=30) and testing cohorts (n=21) are summarized in **Table 1**. All patients in the training cohort were BCLC-C stage, including 27 patients (90%) with portal vein tumor emboli and other 3 cases with hepatic venous thrombus, while all patients in the testing cohort were BCLC-A (n=18, 86%) and B (n=3, 14%). The testing cohort was characterized by lower AFP (median 23.88 *vs.* 1436.64 ng/ml, P=0.003), AST (median 29.5 *vs.* 59 U/L, P<0.001), LDH (median 248 *vs.* 148 U/L, P<0.001) and APTT (30.5 *vs.* 34.5 s, P= 0.007), more patients infected with HBV (60% *vs.* 90%, P=0.016) while fewer patients with hypoalbuminemia (36.4 *vs.* 32.8, P=0.002). Patients in the testing cohort had lower normal liver volume (median 758.75 *vs.* 948.32 ml, P<0.001) than those in the training cohort. Dose-volumetric parameters, including the volume of GTV/CTV1 (P<0.001). Vs5 (p =0.024), Vs40 (P=0.041), SVs5 (P=0.016), SVs30 (P=0.044), SVs35 (P=0.014), and SVs40 (P=0.008) differed significantly between the two groups, with no significant difference in the remaining parameters.

**Table 1:** Information of patients in different treatment groups.

Factor	Level	Training group	Testing group	P-value
Number of patients		30	21	
liver regeneration	No	18 (60%)	8 (38%)	0.12
	Yes	12 (40%)	13 (62%)	
Gender	female	2 (7%)	3 (14%)	0.37
	male	28 (93%)	18 (86%)	
Age, median (IQR), yrs		48 (41, 58)	47 (39, 54)	0.65
Height, median (IQR), cm		165 (162, 170)	165 (160, 170)	0.66
Weight, median (IQR), kg		57.5 (51, 70)	57 (52, 62)	0.61
GTV/CTV1 dose, Gy	45	2 (6%)	0 (0%)	0.037
	50	3 (10%)	11 (52%)	
	56	2 (7%)	0 (0%)	
	60	23 (77%)	10 (48%)	
Fractions	12	1 (3%)	0 (0%)	<0.001
	15	12 (40%)	0 (0%)	
	20	17 (57%)	4 (20%)	
	25	0 (0%)	17 (80%)	
Tumor emboli	No	0 (0%)	21 (100%)	<0.001
	Yes	30 (100%)	0 (0%)	
BCLC stage	A	0 (0%)	18 (86%)	<0.001
	B	0 (0%)	3 (14%)	
	C	30 (100%)	0 (0%)	
Tumor size, median (IQR), cm		11.2 (9.3,13.7)	0 (0,0)	<0.001
Child-Pugh	A	25 (83.3%)	20 (95.2)	0.381
	B7	5 (16.7%)	1(4.8)	
hepatitis B surface antigen	positive	29 (96.7%)	19 (90%)	0.86
	negative	1 (3.3%)	2 (10%)	
AFP, median (IQR), ng/mL		1436.64 (65.67, 8446.97)	23.875 (2.9, 298.69)	0.003
RBC, median (IQR), ×10^12^/L		4.385 (3.85, 4.75)	4.3 (4.04, 4.77)	0.87
HGB, median (IQR), g/L		129.5 (115, 141)	125 (121, 136)	0.86
PLT, median (IQR), ×10^9^/L		186.5 (135, 252)	204 (175, 275)	0.54
Tbil, median (IQR), umol/L		15.9 (10.4, 23.2)	10.4 (7.9, 15.8)	0.053
ALT, median (IQR), u/L		46 (28, 66)	36 (24, 52)	0.31
Albumin, median (IQR), g/L		32.85 (30.5, 34.4)	36.4 (34.6, 38.2)	0.002
AST, median (IQR), u/L		59.5 (44, 97)	29 (29, 37)	<0.001
LDH, median (IQR)		248 (207, 360)	148 (139, 175)	<0.001
APTT, median (IQR)		34.5 (31, 38)	30.5 (27.4, 32.8)	0.007
GTV/CTV1 volume, median (IQR), mL		829.77 (556.25, 1395.66)	54.91 (41.82, 86.39)	<0.001
Liver volume, median (IQR), mL		758.75 (610.73, 1008.79)	943.32 (852.15, 1125.03)	0.026
Liver-Dmean, median (IQR), Gy		15.2 (12.5, 19.5)	17.6 (13.3, 20.7)	0.44
Dataset 1: Vx(%)				
V5, median (IQR)		68.07 (58.92, 82.65)	64.01 (46.97, 78.52)	0.13
V10, median (IQR)		40.99 (34.36, 53.16)	53.16 (32.51, 63.57)	0.69
V15, median (IQR)		29.095 (23.26, 45.74)	39.83 (27.6, 51.3)	0.23
V20, median (IQR)		24.08 (17.46, 38.55)	33.94 (23.91, 41.81)	0.12
V25, median (IQR)		20.71 (14.03, 29.82)	27.31 (20.95, 37.87)	0.072
V30, median (IQR)		17.445 (11.69, 25.23)	24.92 (18.03, 30.24)	0.088
V35, median (IQR)		15.16 (9.8, 19.99)	20.72 (13.51, 23.17)	0.11
V40, median (IQR)		12.51 (7.52, 16)	15.36 (11.3, 19.65)	0.15
Dataset 2: VsX(mL)				
Vs5, median (IQR)		231.281 (106.039, 340.5)	369.948 (226.726, 451.895)	0.024
Vs10, median (IQR)		440.61 (301.988, 540.796)	476.129 (328.414, 567.966)	0.24
Vs15, median (IQR)		494.219 (408.691, 660.603)	595.253 (441.678, 645.371)	0.29
Vs20, median (IQR)		576.736 (441.247, 726.929)	642.055 (500.469, 757.203)	0.23
Vs25, median (IQR)		611.642 (464.236, 768.908)	683.681 (559.181, 818.222)	0.15
Vs30, median (IQR)		626.677 (482.953, 802.44)	728.232 (615.237, 911.303)	0.085
Vs35, median (IQR)		640.942 (499.924, 855.575)	766.951 (656.837, 963.722)	0.053
Vs40, median (IQR)		658.308 (522.279, 889.097)	788.962 (686.976, 973.066)	0.041
Dataset 3: SVsX(mL/m^2^)				
SVs5, median (IQR)		147.976 (55.9661, 204.89)	238.205 (137.36, 305.654)	0.016
SVs10, median (IQR)		257.066 (187.943, 305.571)	308.596 (210.072, 409.522)	0.14
SVs15, median (IQR)		298.816 (258.908, 380.281)	353.752 (277.593, 457.307)	0.13
SVs20, median (IQR)		341.586 (288.752, 428.616)	394.046 (339.95, 486.134)	0.085
SVs25, median (IQR)		354.403 (309.701, 480.15)	431.839 (350.927, 513.324)	0.056
SVs30, median (IQR)		361.757 (316.607, 516.365)	471.309 (384.163, 534.623)	0.044
SVs35, median (IQR)		374.189 (323.059, 528.796)	502.75 (418.129, 570.836)	0.014
SVs40, median (IQR)		385.501 (332.418, 542.123)	526.548 (440.686, 602.561)	0.008

HBV, hepatitis B virus; RBC, red blood cells count; HGB, hemoglobin; PLT, platelets; Tbil, total bilirubin; ALTa, lanine aminotransferase; AST, aspartate aminotransferase; LDH, lactic dehydrogenase; APTT, activated partial thromboplastin time ; AFP, alpha-fetoprotein. Dataset 1: the percentage of normal protected liver volume (%) spared from at least x Gy (Vx); Dataset 2: the absolute normal liver volume (mL) spared from at least x Gy (Vsx), Dataset 3: standard residual normal liver volume (mL/m2) spared from at least x Gy (SVsx).

### Volumetric Data for Liver Regeneration

**Figure 2:**
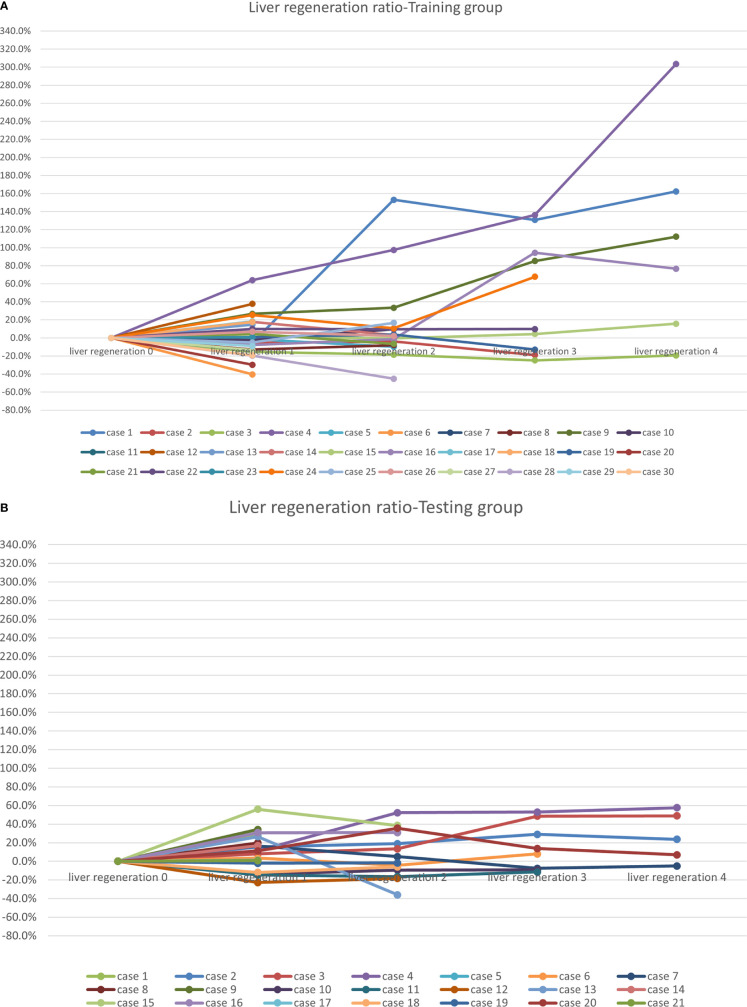
Liver regeneration growth ratio after radiotherapy: **(A)** the training cohort; and **(B)** the testing cohort.

During the observation period of 1 year, a total of 25 patients (49%) experienced liver regeneration in the entire cohort. The change trends of liver regeneration after radiotherapy are shown in **Figures 2A, B**.

In the training cohort of 30 patients, 9 patients underwent surgery after radiotherapy at 3-12 months, and 5 patients at the second evaluation node (liver regeneration 2) experienced tumor progression in the area of interest, and the liver regeneration assessments were therefore stopped. The mean protected hepatic lobe and segment volume before radiotherapy was 605.2 ± 371 mL, increasing to 648.7 ± 345.6 mL after radiotherapy. Liver regeneration occurred in 12 of 30 patients, yielding a liver regeneration rate of 40%. Among them, 6, 1, 2, and 3 patients had liver regeneration rates of 10%–20%, 20–50%, 50%–100%, and greater than 100%, respectively.

In the testing cohort of 21 patients, 4 patients in the testing cohort after the first evaluation node (liver regeneration 1) experienced tumor progression in the area of interest. The mean protected hepatic lobe and segment volume before radiotherapy was 532.5 ± 450.7 mL, increasing to 578.9 ± 412.2 mL after radiotherapy. Liver regeneration occurred in 13 out of 21 patients, yielding a liver regeneration rate of 62%, which was comparable to the rate noted in the training cohort (P =0.12). Among them, 3, 8, 2, and 0 patients had liver regeneration rates of 10%–20%, 20–50%, 50%–100%, and greater than 100%, respectively.

Twenty-four cases of Child-Pugh degradation were detected within 2 weeks after radiotherapy. In the training cohort, 17 patients experienced an increase in Child-Pugh score, including 13 patients with +1 score due to decreased serum albumin levels, 3 patients with +2 scores, and 1 patient with +3 scores. In the validation cohort, 7 patients experienced an increase of one score in Child-Pugh score due to decreased serum albumin levels. There was a negative correlation between the degree of liver regeneration and the increase in Child-Pugh score (P=0.006, analysis of variance). Over a longer observation period, 13 (51.2%) patient’s liver function gradually recovered with the appearance of liver regeneration. Liver regeneration did not occur in the remaining 11 cases, of which 3 patient’s liver function improved spontaneously within 3 months and other 8 cases remained unimproved.

### Risk Group Sub-Classification Based on ALT and SVs20

**Figure 3:**
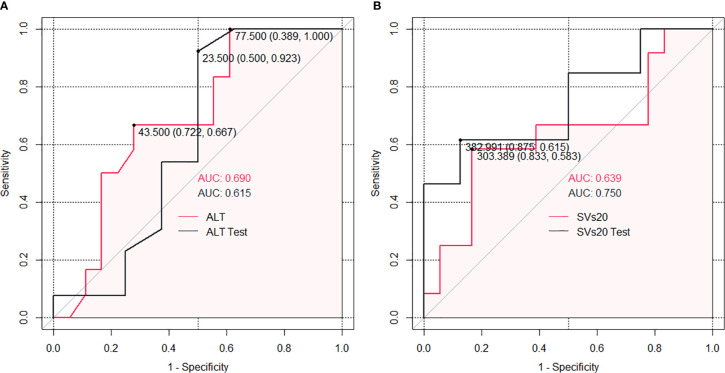
The cut-off points and AUC of each optimal predictors by ROC analysis: **(A)** ALT; **(B)** SVs20.

Datasets 1 and 2 had no significant correlation with liver regeneration. Dataset 3 and clinical factors were used separately for further analysis. According to the subgroup ROC analysis of ALT and SVs20 in the training cohort, the AUC of SVs20 was 0.639 (95% confidence interval [CI], 0.833–0.583) with a cut-off value of 303.4 mL (sensitivity, 65.00%; specificity, 86.15%; **Figure 3A**), and the AUC of ALT was 0.690 (95% CI, 0.722–0.667) with a cutoff value of 43 U/L (sensitivity, 65.00%; specificity, 90.77%; **Figure 3B**). Risk group sub-classification was performed according to the cutoff points. As shown in **Table 2**, a combination of ALT and SVs20 demonstrated clear differentiation in the risk of liver generation between the subgroups in the training cohort (P=0.049) and the entire cohort (P=0.032). The proportion of patients with liver regeneration decreased progressively with 88.9% in the high-probability group (ALT <43 U/L and SVs20 <303.4 mL*/m^2^*), 60% in the high-intermediate probability group (ALT ≥43 U/L and SVs20 <303.4 mL*/m^2^*), 43.75% in the low-intermediate probability group (ALT <43 U/L and SVs20 ≥303.4 mL*/m^2^*), and 33% in the low-probability group (ALT≥43 U/L and SVs20≥303.4 mL*/m^2^*), indicating that a combination of high ALT (≥43 U/L) and high SVs20 (≥303.4 mL*/m^2^*) conferred the greatest risk of poor liver regeneration.

**Table 2:** Risk Group Sub-classification based on ALT and SVs20.

Sub-classification	Training cohort (n=30)			Entire cohort (n=51)		
	NLR, n (%)	LR, n (%)	P	NLR, n (%)	LR, n (%)	P
ALT<43U/L SVs20<303.4 mL/m^2^	1 (16.7%)	5 (83.3%)	0.049	1 (11.1%)	8 (88.9%)	0.032
ALT≥43U/L SVs20<303.4 mL/m^2^	2 (50%)	2 (50%)		2 (40%)	3 (60%)	
ALT<43U/L SVs20≥303.4 mL/m^2^	5 (62.5%)	3 (37.5%)		9 (56.2%)	7 (43.8%)	
ALT≥43U/L SVs20≥303.4 mL/m^2^	10 (83.3%)	2 (16.7%)		14 (66.7%)	7 (33.3%)	

LR, liver regeneration; NLR, no liver regeneration; ALT, alanine aminotransferase; SVs20, standard residual normal liver volume (mL/m2) spared from at least 20Gy.

### Establishment and Assessment of the prognostic Nomogram

**Figure 4:**
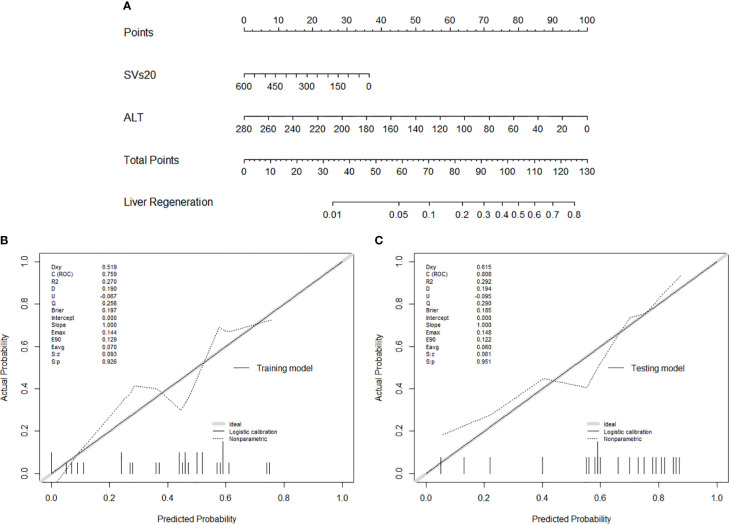
**(A)** Nomogram of liver regeneration based on SVs20 and ALT; **(B)** The calibration curve in the training cohort; **(C)** The calibration curve in the testing cohort.

To provide more convenient for clinical liver regeneration prediction and avoid overfitting, only ALT among pre-radiotherapy laboratory variables and SVs20 among dataset 3 parameters were found to be optimal predictors for liver regeneration modeling. Finally, an SVs20 based nomogram incorporating ALT and SVs20 was established (**Figure 4A**). The model and nomogram showed high predictive accuracy (C-index =0.759, **Figure 4B**) in the training cohort for predicting liver regeneration. The calibration curve confirmed the excellent calibration capability of the model, and the probability predicted by the model was in good agreement with the actual observed values of liver regeneration. The nomogram performed well in external validation (C-index =0.808, **Figure 4C**) with high discriminatory accuracy in the testing cohort (**Supplementary Figure**).

## Discussion

In this study, we designed a new target area delineation to protect the normal liver for regeneration and prospectively evaluate liver regeneration after radiotherapy. After radiotherapy, 40% (12/30) of cases in the preoperative downstaging group and 61.9% (13/21) of cases in the postoperative adjuvant group experienced liver regeneration. We further found that pretreatment ALT and SVs20 were the optimal variables for liver regeneration modeling. The risk stratification model based on the cutoffs of SVs20 (303.4 mL/m^2^) and ALT (43 u/L) was able to effectively discriminate the probability of liver regeneration. The model and nomogram of liver regeneration showed good prediction performance (AUC=0.759) in the training cohort and performed well (AUC=0.808) in the validation cohort, justifying its application values.

In the past decade, adjuvant radiotherapy has been confirmed to provide considerably improved treatment outcomes (19, 21–24). Neoadjuvant radiotherapy provided better overall survival and recurrence-free survival rates compared to surgery alone (20). Yeh et al. showed that 11% of HCCs could become resectable with a 2-year OS rate of 67% and a median survival of 30 months (25). Chong et al. (26) reported that 26 of 98 (26.5%) patients were downstaged after concurrent chemoradiotherapy (CCRT) followed by hepatic arterial infusion chemotherapy (HAIC) and underwent subsequent curative resection. Disease-specific survival was significantly better in the resection group after localized downstaging. Lee et al. (27) also reported that 41 (16.9%) patients underwent curative resection after CCRT followed by HAIC, and the 5-year survival rate in the curative resection group after CCRT improved significantly compared to that of the CCRT alone group (49.6%: 9.8%, P< 0.001). Preoperative downstaging radiotherapy may provide better control of the local tumor and simultaneously promote liver regeneration, creating a better opportunity for wide-margin surgery. However, the insufficient volume of the future liver remnant (FLR) remains a serious constraint that hinders R0–R1 resection (23). Inadequate functional liver volume is also a major cause of liver function deterioration after radiotherapy. Therefore, compensating for the loss of liver mass by regeneration is of great importance, especially in patients with large tumors and limited healthy livers (28). Consequently, a precise radiotherapy plan based on the accurate prediction of liver regeneration is urgently needed in clinical practice.

Previous nomogram models based on clinical factors have focused on risk assessment, such as estimating the risk of radiation-induced liver disease (RILD) and mortality following radiotherapy (29–31). Choi et al. (32) showed that adjuvant radiotherapy with a dose of 40–50 Gy in 20-25 fractions delayed the liver regeneration process after partial hepatectomy. However, nomogram models based on dose-volume metrics for benefit assessment associated with liver regeneration in HCC patients treated with radiotherapy have never been established. We found that liver regeneration was significantly associated with domestic dataset 3(SVs, mL/m^2^), but not with dataset 1 (V, %) and dataset 2 (Vs, mL). The current study identified, for the first time, SVs20 as a key predictor for liver generation following radiotherapy with an optimal cutoff of 303.4, suggesting that fewer constraints on the dose of residual liver should be taken into account to promote liver regeneration. Meanwhile, the risk of RILD following dose escalation is a major cause of liver failure after radiotherapy, and stricter dose-limiting to the functional liver should be prioritized in radiation planning. Based on our past experience of hepatic toxicity following radiotherapy, V20 (dataset 1) in normal liver volume of 48.5% as the liver tolerance predicted RILD risks well in primary liver carcinoma patients with Child–Pugh grade A cirrhosis after hypo-fractionated radiotherapy (15). We further found that Vs10 ≥416.2 mL (dataset 2) predicted a progression of at least 1 and Vs10≥621.8 mL of at least 2 points decreased in the Child–Pugh score after stereotactic body radiation therapy (14), highlighting the necessity for hepatotoxicity mitigating in high-risk functional liver areas. Functional liver avoidance with V20 <48.5% and/or Vs10 ≥416.2 mL may be used as a radiotherapy reference in clinical practice by balancing the pros and cons.

Among the pre-RT laboratory variables, ALT was found to have a significant correlation with liver regeneration. ALT is well recognized as a marker of liver injury related to liver parenchymal injury. In line with our current results, Mohapatra et al. (33) reported that a high level of ALT predicted poor liver regeneration following donor hepatectomy. Caldez et al. (34) showed that ALT is hyperactivated when liver cells fail to divide during liver regeneration, indicating that ALT not only acts as a signal of liver damage but is also a crucial metabolic regulator necessary to support tissue recovery. Kimura et al. (35) reported that serum ALT levels after partial liver resection are negatively correlated with L-ascorbic acid and L-ascorbic acid 2-Glucoside, which stimulates liver regeneration. Similarly, Lin et al. (36) revealed the crucial role of translationally controlled tumor protein in liver regeneration, as well as enhancing the recovery of ALT after liver resection in humans. Ito et al. (37) found that partially hepatectomized rats had lower serum ALT levels and higher recovery of remnant liver weight. Although the precise mechanisms are not fully understood, these results strongly indicate that lower ALT levels are associated with better liver regeneration. This potential mechanism requires further exploration and research.

Our study has several limitations. First, this study was conducted in China, where HBV-associated HCC rates are high. The applicability of this nomogram for patient cohorts in other areas is uncertain and requires further validation. Second, the sample size was small in this study, the reliability of the model has been verified in two different prospective studies, and it is still worth verifying with a larger sample size in the future.

In conclusion, this simple-to-use nomogram incorporating ALT and SVs20 is beneficial to the constraints of the normal liver outside the radiotherapy target area. It may provide a reference for clinicians to make prognosis-based decisions without complex calculations. Further validation with multicenter data is warranted to verify its practicability in patients with HCC who have undergone radiotherapy.

## Data Availability Statement

The datasets presented in this article are not readily available because the confidentiality rules of hospital. Requests to access the datasets should be directed to sutingshi@163.com.

## Ethics Statement

The studies involving human participants were reviewed and approved by Institutional Ethics Committee of Guangxi Medical University Cancer Hospital (LW2021009). The patients/participants provided their written informed consent to participate in this study.

## Author Contributions

Conception and design: T-SS. Administrative support: T-SS, S-XL, and L-QuL. Provision of study materials or patients: All authors. Collection and assembly of data: L-QiL. Data analysis and interpretation: T-SS and L-QiL. Manuscript writing: T-SS and L-QiL. All authors contributed to the article and approved the submitted version.

## Funding

This research was supported by the National Natural Science Foundation of China (81903257 and 81960534), and Guangxi Natural Science Foundation (CN) (2020GXNSFAA297171), and Cancer Precision Radiotherapy Spark Program of China International Medical Foundation (2019-N-11-01), High-level innovation team and outstanding scholar program in Guangxi Colleges and Universities, and Guangxi Medical University Training Program for Distinguished Young Scholars, and Guangxi BaGui Scholars’ Special Fund.

## Conflict of Interest

The authors declare that the research was conducted in the absence of any commercial or financial relationships that could be construed as a potential conflict of interest.

## Publisher’s Note

All claims expressed in this article are solely those of the authors and do not necessarily represent those of their affiliated organizations, or those of the publisher, the editors and the reviewers. Any product that may be evaluated in this article, or claim that may be made by its manufacturer, is not guaranteed or endorsed by the publisher.

## Supplementary Material

Supplementary Figure The AUC of the training and testing model using ROC analysis.

## References

[1] Yang JD Hainaut P Gores GJ Amadou A Plymoth A Roberts LR. A Global View of Hepatocellular Carcinoma: Trends, Risk, Prevention and Management. Nat Rev Gastroenterol Hepatol (2019) 16(10):589–604. 10.1038/s41575-019-0186-y 31439937 PMC6813818

[2] Su TS Li LQ Meng WW Wang YD Chen YT Li JX. Long-Term Survival Analysis of Transarterial Chemoembolization Plus Radiotherapy *vs.* Radiotherapy for Hepatocellular Carcinoma With Macroscopic Vascular Invasion. Front Oncol (2020) 10:1205. 10.3389/fonc.2020.01205 32850352 PMC7416768

[3] Su TS Liang P Liang J Lu HZ Jiang HY Cheng T. Long-Term Survival Analysis of Stereotactic Ablative Radiotherapy Versus Liver Resection for Small Hepatocellular Carcinoma. Int J Radiat Oncol Biol Phys (2017) 98(3):639–46. 10.1016/j.ijrobp.2017.02.095 28581406

[4] Sun J Wang Q Hong ZX Li WG He WP Zhang T. Stereotactic Body Radiotherapy Versus Hepatic Resection for Hepatocellular Carcinoma (≤ 5 Cm): A Propensity Score Analysis. Hepatol Int (2020) 14(5):788–97. 10.1007/s12072-020-10088-0 32886334

[5] Yoon SM Ryoo BY Lee SJ Kim JH Shin JH An JH. Efficacy and Safety of Transarterial Chemoembolization Plus External Beam Radiotherapy vs Sorafenib in Hepatocellular Carcinoma With Macroscopic Vascular Invasion: A Randomized Clinical Trial. JAMA Oncol (2018) 4(5):661–9. 10.1001/jamaoncol.2017.5847 PMC5885246 29543938

[6] Li LQ Zhou Y Huang Y Liang P Liang SX Su TS. Stereotactic Body Radiotherapy Versus Intensity-Modulated Radiotherapy for Hepatocellular Carcinoma With Portal Vein Tumor Thrombosis. Hepatol Int (2021) 15(3):630–41. 10.1007/s12072-021-10173-y 33818714

[7] Vogel A Cervantes A Chau I Daniele B Llovet J Meyer T. Hepatocellular Carcinoma: ESMO Clinical Practice Guidelines for Diagnosis, Treatment and Follow-Up. Ann Oncol (2018) 29(Supplement_4):iv238–iv55. 10.1093/annonc/mdy308 30285213

[8] Benson AB 3rd D'Angelica MI Abbott DE Abrams TA Alberts SR Saenz DA. NCCN Guidelines Insights: Hepatobiliary Cancers, Version 1.2017. J Natl Compr Canc Netw (2017) 15(5):563–73. 10.6004/jnccn.2017.0059 PMC5557008 28476736

[9] Lu J Zhang XP Zhong BY Lau WY Madoff DC Davidson JC. Management of Patients With Hepatocellular Carcinoma and Portal Vein Tumour Thrombosis: Comparing East and West. Lancet Gastroenterol Hepatol (2019) 4(9):721–30. 10.1016/S2468-1253(19)30178-5 31387735

[10] Korean Liver Cancer A National Cancer Center GK. 2018 Korean Liver Cancer Association-National Cancer Center Korea Practice Guidelines for the Management of Hepatocellular Carcinoma. Korean J Radiol (2019) 20(7):1042–113. 10.3348/kjr.2019.0140 PMC6609431 31270974

[11] Cheng SQ Chen MS Cai JQ Sun JX Guo RP Bi XY. Chinese Expert Consensus on Multidisciplinary Diagnosis and Treatment of Hepatocellular Carcinoma With Portal Vein Tumor Thrombus (2018 Edition). Liver Cancer (2020) 9(1):28–40. 10.1159/000503685 32071907 PMC7024893

[12] Zhou J Sun HC Wang Z Cong WM Wang JH Zeng MS. Guidelines for Diagnosis and Treatment of Primary Liver Cancer in China (2017 Edition). Liver Cancer (2018) 7(3):235–60. 10.1159/000488035 PMC6167671 30319983

[13] Liang SX Zhu XD Xu ZY Zhu J Zhao JD Lu HJ. Radiation-Induced Liver Disease in Three-Dimensional Conformal Radiation Therapy for Primary Liver Carcinoma: The Risk Factors and Hepatic Radiation Tolerance. Int J Radiat Oncol Biol Phys (2006) 65(2):426–34. 10.1016/j.ijrobp.2005.12.031 16690430

[14] Su TS Luo R Liang P Cheng T Zhou Y Huang Y. A Prospective Cohort Study of Hepatic Toxicity After Stereotactic Body Radiation Therapy for Hepatocellular Carcinoma. Radiother Oncol (2018) 129(1):136–42. 10.1016/j.radonc.2018.02.031 29548558

[15] Liang SX Huang XB Zhu XD Zhang WD Cai L Huang HZ. Dosimetric Predictor Identification for Radiation-Induced Liver Disease After Hypofractionated Conformal Radiotherapy for Primary Liver Carcinoma Patients With Child-Pugh Grade A Cirrhosis. Radiother Oncol (2011) 98(2):265–9. 10.1016/j.radonc.2010.10.014 21056489

[16] Gong WF Zhong JH Lu Z Zhang QM Zhang ZY Chen CZ. Evaluation of Liver Regeneration and Post-Hepatectomy Liver Failure After Hemihepatectomy in Patients With Hepatocellular Carcinoma. Biosci Rep (2019) 39(8):BSR20190088. 10.1042/BSR20190088 31383787 PMC6706596

[17] Wang Z Peng Y Hu J Wang X Sun H Sun J. Associating Liver Partition and Portal Vein Ligation for Staged Hepatectomy for Unresectable Hepatitis B Virus-Related Hepatocellular Carcinoma: A Single Center Study of 45 Patients. Ann Surg (2020) 271(3):534–41. 10.1097/SLA.0000000000002942 29995681

[18] Pourcher G El-Kehdy H Kanso F Groyer-Picard MT Gaillard M Trassard O. Volumetric Portal Embolization: A New Concept to Improve Liver Regeneration and Hepatocyte Engraftment. Transplantation (2016) 100(2):344–54. 10.1097/TP.0000000000001024 26757049

[19] Bai T Chen J Xie ZB Wu FX Wang SD Liu JJ. The Efficacy and Safety of Postoperative Adjuvant Transarterial Embolization and Radiotherapy in Hepatocellular Carcinoma Patients With Portal Vein Tumor Thrombus. Onco Targets Ther (2016) 9:3841–8. 10.2147/OTT.S104307 PMC4930237 27390524

[20] Wei X Jiang Y Zhang X Feng S Zhou B Ye X. Neoadjuvant Three-Dimensional Conformal Radiotherapy for Resectable Hepatocellular Carcinoma With Portal Vein Tumor Thrombus: A Randomized, Open-Label, Multicenter Controlled Study. J Clin Oncol (2019) 37(24):2141–51. 10.1200/JCO.18.02184 PMC6698917 31283409

[21] Sun J Yang L Shi J Liu C Zhang X Chai Z. Postoperative Adjuvant IMRT for Patients With HCC and Portal Vein Tumor Thrombus: An Open-Label Randomized Controlled Trial. Radiother Oncol (2019) 140:20–5. 10.1016/j.radonc.2019.05.006 31176205

[22] Yu W Wang W Rong W Wang L Xu Q Wu F. Adjuvant Radiotherapy in Centrally Located Hepatocellular Carcinomas After Hepatectomy With Narrow Margin (<1 Cm): A Prospective Randomized Study. J Am Coll Surg (2014) 218(3):381–92. 10.1016/j.jamcollsurg.2013.11.030 24559953

[23] Wang WH Wang Z Wu JX Zhang T Rong WQ Wang LM. Survival Benefit With IMRT Following Narrow-Margin Hepatectomy in Patients With Hepatocellular Carcinoma Close to Major Vessels. Liver Int (2015) 35(12):2603–10. 10.1111/liv.12857 25939444

[24] Rong W Yu W Wang L Wu F Zhang K Chen B. Adjuvant Radiotherapy in Central Hepatocellular Carcinoma After Narrow-Margin Hepatectomy: A 10-Year Real-World Evidence. Chin J Cancer Res (2020) 32(5):645–53. 10.21147/j.issn.1000-9604.2020.05.09 PMC7666779 33223759

[25] Yeh SA Chen YS Perng DS. The Role of Radiotherapy in the Treatment of Hepatocellular Carcinoma With Portal Vein Tumor Thrombus. J Radiat Res (2015) 56(2):325–31. 10.1093/jrr/rru104 PMC4380051 25411553

[26] Chong JU Choi GH Han DH Kim KS Seong J Han KH. Downstaging With Localized Concurrent Chemoradiotherapy Can Identify Optimal Surgical Candidates in Hepatocellular Carcinoma With Portal Vein Tumor Thrombus. Ann Surg Oncol (2018) 25(11):3308–15. 10.1245/s10434-018-6653-9 30083834

[27] Lee HS Choi GH Choi JS Kim KS Han KH Seong J. Surgical Resection After Down-Staging of Locally Advanced Hepatocellular Carcinoma by Localized Concurrent Chemoradiotherapy. Ann Surg Oncol (2014) 21(11):3646–53. 10.1245/s10434-014-3652-3 24916746

[28] Wei W Hua C Zhang T Dirsch O Gremse F Homeyer A. Size of Portally Deprived Liver Lobe After Portal Vein Ligation and Additional Partial Hepatectomy: Result of Balancing Proliferation and Apoptosis. Sci Rep (2020) 10(1):4893. 10.1038/s41598-020-60310-0 32184404 PMC7078252

[29] Velec M Haddad CR Craig T Wang L Lindsay P Brierley J. Predictors of Liver Toxicity Following Stereotactic Body Radiation Therapy for Hepatocellular Carcinoma. Int J Radiat Oncol Biol Phys (2017) 97(5):939–46. 10.1016/j.ijrobp.2017.01.221 28333016

[30] Huang WY Tsai CL Que JY Lo CH Lin YJ Dai YH. Development and Validation of a Nomogram for Patients With Nonmetastatic BCLC Stage C Hepatocellular Carcinoma After Stereotactic Body Radiotherapy. Liver Cancer (2020) 9(3):326–37. 10.1159/000505693 PMC7325119 32647634

[31] Zhang L Yan L Niu H Ma J Yuan BY Chen YH. A Nomogram to Predict Prognosis of Patients With Unresected Hepatocellular Carcinoma Undergoing Radiotherapy: A Population-Based Study. J Cancer (2019) 10(19):4564–73. 10.7150/jca.30365 PMC6746140 31528220

[32] Choi JH Kim K Chie EK Jang JY Kim SW Oh DY. Does Adjuvant Radiotherapy Suppress Liver Regeneration After Partial Hepatectomy? Int J Radiat Oncol Biol Phys (2009) 74(1):67–72. 10.1016/j.ijrobp.2008.06.1941 18963543

[33] Mohapatra N Sinha PK Sasturkar SV Patidar Y Pamecha V. Preoperative Alanine Aminotransferase and Remnant Liver Volume Predict Liver Regeneration After Live Donor Hepatectomy. J Gastrointest Surg (2020) 24(8):1818–26. 10.1007/s11605-019-04332-8 31388890

[34] Caldez MJ Van Hul N Koh HWL Teo XQ Fan JJ Tan PY. Metabolic Remodeling During Liver Regeneration. Dev Cell (2018) 47(4):425–38.e5. 10.1016/j.devcel.2018.09.020 30344111

[35] Kimura M Moteki H Uchida M Natsume H Ogihara M. L-Ascorbic Acid- and L-Ascorbic Acid 2-Glucoside Accelerate In Vivo Liver Regeneration and Lower Serum Alanine Aminotransaminase Activity in 70% Partially Hepatectomized Rats. Biol Pharm Bull (2014) 37(4):597–603. 10.1248/bpb.b13-00839 24818255

[36] Lin Z Zhang X Wang J Liu W Liu Q Ye Y. Translationally Controlled Tumor Protein Promotes Liver Regeneration by Activating Mtorc2/AKT Signaling. Cell Death Dis (2020) 11(1):58. 10.1038/s41419-020-2231-8 31974368 PMC6978394

[37] Ito K Ozasa H Noda Y Koike Y Arii S Horikawa S. Splenic Artery Ligation Improves Remnant Liver Function in Partially Hepatectomized Rats With Ischemia/Reperfusion Injury. Liver Int (2007) 27(3):400–7. 10.1111/j.1478-3231.2006.01432.x 17355463

