# Supplementary figures and images for: A Prospective Study of Liver Regeneration After Radiotherapy Based on a New (Su’S) Target Area Delineation
Source: Front Oncol. 2021 Aug 26;11:680303. doi: 10.3389/fonc.2021.680303 (PMC8426619; doi:10.3389/fonc.2021.680303)

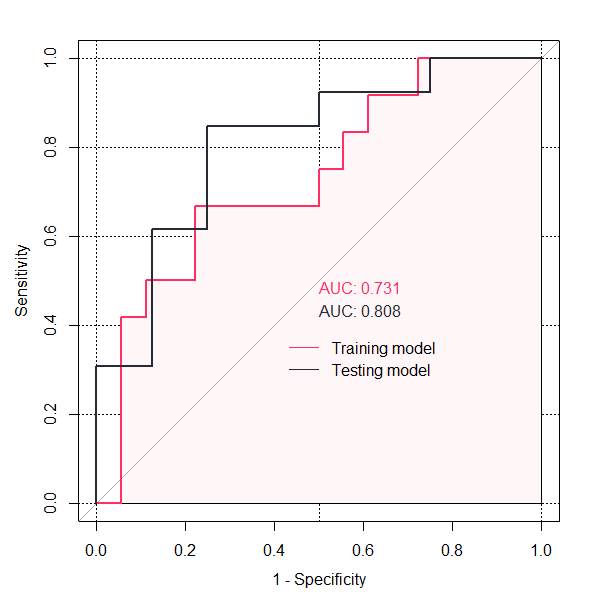

Supplement: Supplementary Figure — The AUC of the training and testing model using ROC analysis. [file Image_1.tiff]
